# Mice Genetically Depleted of Brain Serotonin Display Social Impairments, Communication Deficits and Repetitive Behaviors: Possible Relevance to Autism

**DOI:** 10.1371/journal.pone.0048975

**Published:** 2012-11-06

**Authors:** Michael J. Kane, Mariana Angoa-Peréz, Denise I. Briggs, Catherine E. Sykes, Dina M. Francescutti, David R. Rosenberg, Donald M. Kuhn

**Affiliations:** 1 Research and Development Service, John D. Dingell VA Medical Center, Detroit, Michigan, United States of America; 2 Department of Psychiatry and Behavioral Neurosciences, Wayne State University School of Medicine, Detroit, Michigan, United States of America; University of South Florida, United States of America

## Abstract

Autism is a complex neurodevelopmental disorder characterized by impaired reciprocal social interaction, communication deficits and repetitive behaviors. A very large number of genes have been linked to autism, many of which encode proteins involved in the development and function of synaptic circuitry. However, the manner in which these mutated genes might participate, either individually or together, to cause autism is not understood. One factor known to exert extremely broad influence on brain development and network formation, and which has been linked to autism, is the neurotransmitter serotonin. Unfortunately, very little is known about how alterations in serotonin neuronal function might contribute to autism. To test the hypothesis that serotonin dysfunction can contribute to the core symptoms of autism, we analyzed mice lacking brain serotonin (via a null mutation in the gene for tryptophan hydroxylase 2 (TPH2)) for behaviors that are relevant to this disorder. Mice lacking brain serotonin (TPH2−/−) showed substantial deficits in numerous validated tests of social interaction and communication. These mice also display highly repetitive and compulsive behaviors. Newborn TPH2−/− mutant mice show delays in the expression of key developmental milestones and their diminished preference for maternal scents over the scent of an unrelated female is a forerunner of more severe socialization deficits that emerge in weanlings and persist into adulthood. Taken together, these results indicate that a hypo-serotonin condition can lead to behavioral traits that are highly characteristic of autism. Our findings should stimulate new studies that focus on determining how brain hyposerotonemia during critical neurodevelopmental periods can alter the maturation of synaptic circuits known to be mis-wired in autism and how prevention of such deficits might prevent this disorder.

## Introduction

Autism is a neurodevelopmental disorder characterized behaviorally by three main core symptoms- impaired reciprocal social interactions, communication deficits and repetitive behaviors. The mechanisms underlying autism are complex and likely involve alterations in synaptic development and signaling, neurotrophic gene function and neurotransmission. In fact, at the clinical level, the extensive heterogeneity seen across core symptoms has led to an evolution in terminology from autism to autism spectrum disorders (ASDs). ASDs include autistic disorder (i.e., “classic” autism), Asperger Syndrome, and Pervasive Developmental Disorder- Not Otherwise Specified (PDD-NOS). ASDs have been associated with more than 440 identified disease genes and 44 genomic loci [1], [2], [3], [4]. Of those cases that can be tied to genetic causes, copy number variants account for 7–20%, 5–7% are attributable to rare single-gene disorders and less than 5% arise from metabolic disorders, leaving about 70% for which a cause cannot yet be identified [5]. The CDC has estimated that ∼1 in 88 children are diagnosed with ASD each year, more than are diagnosed annually with AIDS, diabetes and cancer combined (Autism Speaks; www.autismspeaks.org). This disorder represents an annual cost to our society of about $35 billion in direct and indirect care costs [6].

Defects in function within the serotonin (5HT) neuronal system have long been suspected of involvement in ASD [7], [8], [9], [10]. Perhaps the earliest implication of altered 5HT function in ASD was published by Freedman and colleagues who showed significant elevations in blood 5HT in adults with ASD [11]. This observation fueled thinking that ASD was linked to a platelet “hyperserotonemia” but it was shown subsequently to reflect increased uptake of the amine into platelets, not an increase in “free” 5HT in blood. Further study has shown that individuals with ASD have decreased levels of 5HT receptor binding in brain [12]. Short term reduction in the levels of tryptophan, the precursor of 5HT, exacerbates repetitive, compulsive behaviors (e.g., flapping, rocking, whirling) in drug-free adults with ASD [13]. Children with ASD also show decreases in 5HT synthesis in brain areas important for language production and sensory integration [10]. Polymorphisms in the gene for tryptophan hydroxylase 2 (TPH2), the initial and rate-limiting enzyme in the synthesis of 5HT, have been associated with ASD and repetitive behaviors [14] in some studies [15], [16], [17] while others find no such association [18], [19], [20], [21]. Drugs that target the 5HT neuronal system have been used to treat ASDs [22], [23], [24], [25] but not always with clear efficacy [26]. Taken together, numerous converging lines of evidence suggest that a reduction in function within the 5HT system could contribute to ASDs. However, like most other susceptibility genes and risk factors, 5HT deficits most likely occur in a subset of individuals with ASDs.

In addition to identified deficits in 5HT neurochemical function in ASD discussed above, alterations in 5HT neurochemistry can exert an extremely important influence on the development of the brain. Among monoamine neurotransmitters, 5HT was perhaps the first to be suspected of playing a brain developmental role [27], [28], [29]. 5HT neurons are generated at very early embryonic time points and the 5HT neuronal system is one of the most widely distributed in brain. Developing axons can store and release 5HT before synapses are formed without having the ability to synthesize 5HT [30]. Therefore, alterations in 5HT function during critical neurodevelopmental periods [31], [32] could have profound effects on postnatal CNS function and long-term neurobiological outcome in light of the extremely important roles played by 5HT in synaptogenesis, corticogenesis and network formation in thalamocortical pathways [30], [33], [34], [35], [36], [37], [38]. In fact, the influence of 5HT on the correct allocation of neurons within cortical laminae and columns is so important for normal brain function, disruptions in 5HT homeostasis could underlie various debilitating neuropsychiatric disorders including ASDs [30], [34], [35], [38], [39], [40].

In light of the powerful influence exerted by 5HT on brain development and its suspected role in ASDs, it is surprising that relatively little research has been directed at assessing how 5HT dysfunction may determine susceptibility to this neurodevelopmental disorder. For example, a large number of groups have independently developed rodents with null mutations in the gene for TPH2 [41], [42], [43], [44], [45], [46], [47], [48], [49], [50], [51] but only three of these have reported behaviors in these mice that relate to ASD [41], [48], [51]. Animals lacking the gene for the 5HT transporter (SERT; [52]) or expressing a SERT gain-of-function coding variant seen in ASD [53] show social impairments, and targeted disruption of the 5HT1C receptor gene results in compulsive behaviors [54]. We show presently that mice (young and adult) lacking brain 5HT constitutively via a null mutation in the gene for TPH2 display numerous core behavioral features of ASDs including severely impaired social interactions and communication, and repetitive behaviors. These results should rekindle interest in the study of how brain hyposerotonemia can contribute to ASDs.

## Materials and Methods

### Animals

TPH2−/− mice were generated by deleting exon 1 of the TPH2 gene as described [47]. These mice have no brain TPH2 protein and they express no other compensatory enzymatic or chemical mechanism to hydroxylate tryptophan so their brains are devoid of 5HT and 5-hydroxyindoleacetic acid (5HIAA). 5HT neurons and processes remain intact in TPH2−/− mice and show normal expression of the SERT and 5HT receptors [47], [48]. Wild-type (WT) and TPH2−/− mice used in this study were derived from matings of heterozygous (TPH2+/−) males and heterozygous (Tph2+/−) females on a mixed C57BL/6-Sv129 background. Heterozygous TPH2+/− mice have the same brain levels of 5HT, SERT, and TPH2 as WTs, and were not tested presently. Offspring were housed with their mothers until weaning at PND21 and thereafter litters were housed together for an additional 1 week. Litters were then separated by sex and males and females were housed as groups in 27×48×20 cm cages. All adult mice and weanlings had food and water available ad libitum in home cages. In order to avoid possible confounding effects of litter oversampling, mice of both sexes and genotypes were taken in equal numbers from litters, to the extent possible. Results of all tests reflect groups of mice from at least 3–4 independent litters. Separate cohorts of pups (postnatal days (PND) 1–21), weanlings (PND 23–28), and adult mice (10–12 weeks of age) were acclimated to the behavioral testing room for 1 hr prior to all testing (10am-4pm daily). For studies involving adult mice, independent groups were used for each behavioral test and experimenters scoring behaviors were blind to genotype. For studies involving weanling mice, five unique cohorts were subjected to 1–2 behavioral tests each, with tests separated by 24 hrs: Cohort 1: marble burying and locomotor activity; Cohort 2: light-dark box and nestlet shredding; Cohort 3: social memory and compulsive digging; Cohort 4: novel object test and Cohort 5: social olfactory discrimination. This study was carried out in strict accordance with the recommendations in the Guide for the Care and Use of Laboratory Animals of the National Institutes of Health. The protocol was approved by the Institutional Care and Use Committee of Wayne State University (Permit Number A3310-01).

### Developmental milestones and brain to body weight ratios

Development of selected neonatal reflexes and growth milestones was evaluated as previously described [55], [56]. Briefly, beginning on PND 1, newborn mice were examined daily for acquisition of the following somatosensory reflexes and neurodevelopmental markers: 1) surface righting, between PND 1–13, pups placed on their back must turn over so that all four paws touch the table surface; 2) air righting, between PND 8–21, pups released upside-down from a height of approximately 30 cm must turn right side up and land on all four paws on a bed of litter; 3) forelimb grasping, between PND 4–14, pups must grasp a thin rod with their forepaws and remain suspended for at least 1 second; and 4) negative geotaxis, between PND 1–21, pups placed head down on a 45° incline must turn and start to crawl up the slope. Reflexes were considered acquired only after they had been observed for 2 consecutive days. Physical developmental milestones were examined by assessing the following: 1) pinnae detachment, between PND 1–4; 2) eye opening, between PND 8–21; and 3) fur coloration, between PND 1–8. The method for determination of brain and body weights and brain to body weight ratios is provided in Materials and Methods S1.

### Tests of sociability

#### Maternal scent choice

Maternal scent preference [57] was conducted on PND 14 pups in a transparent polycarbonate cage (19×29×13 cm). The left third of the test cage was filled to a depth of 3 cm with litter from the mother's cage, the center third contained clean litter, and the right third contained litter from the cage of a stranger dam. The position of the test litters (mother and stranger) was alternated across subjects to control for any side preferences. Three 1 min trials, with inter-trial intervals of 10 sec, were administered for each pup. For the first trial, pups were placed in the center of the fresh litter facing the back wall of the test cage. For the second trial, pups were placed in the center of the fresh litter facing the section containing its mother's cage litter. For the third trial, pups faced the section containing the litter of the stranger dam. Time spent in each section of the cage was recorded and averaged across the three trials. The pup was considered to be inside a section when all 4 paws were touching the litter within the specified region.

#### Social memory

Social memory testing was carried out as described [58] and is comprised of 3 habituation trials followed by a single dishabituation trial. Socialized mice (e.g. group housed littermates) were individually placed into a transparent polycarbonate cage (27×48×20 cm) devoid of litter and containing an empty grid enclosure (Ugo Basile, Collegeville, PA, Cat #46503-003) located on one side for 30 min. The location of the grid enclosure was alternated across subjects. Following habituation, a socialized conspecific mouse (WT, same age and sex, but from a different litter) was placed into the grid enclosure and the resident mouse was allowed to interact with the caged intruder. The grid enclosures allowed for social interaction between the two mice and were heavy enough so as not to be moved. In trial 1, latency to investigate and the total time spent investigating the intruder was recorded for 10 min. The caged intruder was removed to its home cage for 10 min and then returned to the cage in a grid enclosure with the resident mouse for 10 min (trial 2). The same procedure was repeated for an additional trial (trial 3). After trial 3, the caged intruder was removed and the grid enclosure was thoroughly cleaned with 70% ethanol to remove any potential olfactory cues. For trial 4, a new intruder (WT, same age and sex as the first intruder, but from a different litter) was put into the grid enclosure and placed into the cage of the resident. Latency to investigate and time spent investigating the new intruder were recorded for 10 min.

#### Novel object test

This test was used as a corollary of restricted interests as previously described [59], [60], [61]. Mice were individually placed into a transparent polycarbonate cage (27×48×20 cm) without litter for 30 min. Following habituation, the mouse was removed briefly from the cage and a novel object (ceramic dish, 6 cm in diameter, 2.25 cm in height) was placed on one side of the cage. The latency to investigate the object (defined as sniffing within 1 cm), total investigation time, and number of contacts with the object were recorded by 2 independent observers. If the animal stood on the object and was not actively investigating (e.g. sniffing), this behavior was not counted as investigation time or as a contact with the object. The position of the novel object within the cage was counterbalanced across subjects.

#### Social olfactory discrimination

Olfactory experiments were adapted from Taylor et al. [62]. Briefly, sponge blocks (2 cm^3^) were individually placed in 50 ml conical tubes containing 2 g of litter from test animals' cage or a different mouse (same sex, age and genotype), and mixed by rocking tubes end over end for 12 hrs. Subjects were placed individually into the test cage (19×29×13 cm) for 30 min and then presented with a block scented with its own bedding and a block scented with bedding from another mouse. Sponge blocks were placed in the two corners of the smallest side of the cage and the positions were counterbalanced across subjects to avoid side preferences. The time spent sniffing (defined as nose less than 1 cm away from the block) or in contact with the block was recorded for a 2 min trial.

#### Direct social interaction

This test was performed as previously described [48] as a modification of the resident intruder test of aggression. Briefly, socialized, adult female mice were housed individually in a transparent polycarbonate cage (19×29 13 cm) for 30 min (“residents”). A conspecific “intruder” (socialized, WT adult female from a different litter) was placed into the cage with the resident. Time spent investigating the intruder (e.g. nose to nose contact, nose to genital contact) by the resident was recorded for 5 min by an experienced observer. Male TPH2−/− mice were not used in tests of direct social interaction because of their extreme aggression [48].

#### Three chamber sociability test

Methods described by Moy and colleagues [63] were followed for this test. The three chamber apparatus, including grid enclosures, was purchased from Ugo Basile (Catalog #46503). The test mouse was acclimated to the empty apparatus for 10 min with access to all 3 chambers. The test mouse was then confined to the middle chamber while an empty grid enclosure was placed into one of the side chambers and a stranger mouse (i.e., stranger 1, a socialized WT mouse, same age and sex from a different litter) inside an identical grid enclosure in the other side chamber. The grid enclosures allowed for social interaction between the two mice and were heavy enough to prevent movement. The location of the grid enclosure and stranger mouse alternated across subjects. To begin a trial, the two middle chamber doors were opened simultaneously for 10 min allowing the subject access to all three chambers. After the trial, the subject mouse was confined to the middle chamber and a new stranger mouse (i.e., stranger 2, same age and sex but from a different litter than stranger 1) was placed inside the grid enclosure and then into the chamber in the position previously occupied by the empty grid enclosure. To begin the next trial, the middle chamber doors were opened simultaneously allowing the subject access to all 3 chambers for 10 min. Time spent in each chamber, the number of entries into each chamber, and total distance travelled were recorded by automated software (EZ Video, AccuScan Systems, Columbus, OH). The time the subject mouse spent sniffing either stranger mouse and/or the grid enclosure was also recorded by an observer as recommended by Fairless et al. [64].

### Social Communication

#### Urine scent marking

Deposition of scent marks as a form of mouse social communication [65] was carried out as previously described [66], [67]. Adult male mice (sexually naïve) were housed individually for 72 hr in transparent polycarbonate cages (19×29×13 cm). On day 1, a WT adult female (sexually naïve) was placed into the cage with the male for 15 min and the animals were allowed to interact freely. On day 2, the cage was lined with filter paper (Whatman Chromatography Paper, 0.34 mm) and 25 µl of 1× PBS was pipetted onto the paper 5 cm from the edge of the cage. Test mice were placed into the cage for 20 minutes and allowed to explore freely. Filter papers were removed from the cages, air-dried for 2 hr, sprayed thoroughly with 0.2% ninhydrin solution in ethanol (Sigma Aldrich, St. Louis, MO) and air-dried overnight. On day 3, cages were lined with fresh filter paper and 25 µl of urine from a WT female in estrous was pipetted onto the paper in the same location as the PBS. The positions of the urine/PBS spots were alternated to avoid side preferences. Mice were placed into the cage for 20 min. The latency to investigate the urine spot and the total time spent investigating it was recorded for the first 5 min. Filter papers were removed and processed as described above. Scent marking behaviors was quantified by placing a transparent grid composed of 1 cm^2^ squares over the ninhydrin-stained filter papers and counting the total number of ninhydrin-stained squares. To analyze urine deposits immediately surrounding the urine spot, the total number of stained squares was counted in a 5 cm^2^ box centered on the urine spot.

### Tests of repetitive/compulsive behaviors

#### Nestlet shredding

This test was performed as previously described [48]. Briefly, pre-weighed nestlets (5×5 cm, Ancare, Bellmore, NY) were placed into a transparent polycarbonate cage (19×29×13 cm) with a single mouse for 60 min. The unshredded remainder of the nestlet was weighed to calculate % nestlet shredded [68].

#### Marble Burying

This test was performed as previously described [48]. Briefly, 20 opaque marbles arrayed in five rows of four were placed on top of 5 cm fresh corncob litter in a transparent polycarbonate cage (27×48×20 cm). The number of marbles with two-thirds of their surface covered were scored as buried during the 30 min test session [68].

#### Compulsive digging

Mice were placed individually into a transparent polycarbonate cage (27×48×20 cm) containing approximately 5 cm of the same corncob bedding used in their home cages. The latency to dig and total time spent digging were measured over the course of 10 min.

### Statistical Analysis

Behavioral tests in PND 1–21 mice were analyzed by student's t-tests. Social memory tests in weanlings and adult mice were first analyzed with a 2-way ANOVA to test for the main effects of genotype and trial and their interaction. In addition, repeated measures ANOVAs were performed to analyze the main effects of trials within and between genotypes. Student's 2-tailed t-tests were performed to analyze the data corresponding to the novel object test, social olfactory discrimination, repetitive behaviors in weanlings, social investigation in adults, three chambers test and latency/time to investigate in the urine-elicited test. Total and 5 cm^2^ grid square counts in the urine scent marking test were analyzed with one-way ANOVA followed by Tukey's multiple comparison. For tests that used males and females as subjects, main effects of genotype and sex were tested for significance using a 2-way ANOVA. When the main effect of sex was not significant, data from males and females was combined for analysis of genotype. p values<0.05 were deemed statistically significant. In those cases where cohorts of mice were used in two tests, Bonferroni's correction was applied to control for the familywise error rate and the p value for significance was set to 0.025. All statistical analyses were carried out using GraphPad Prism version 5.04 for Windows, GraphPad Software, San Diego, CA, www.graphpad.com.

## Results

### Developmental milestones and social recognition in newborn pups

**Figure 1:**
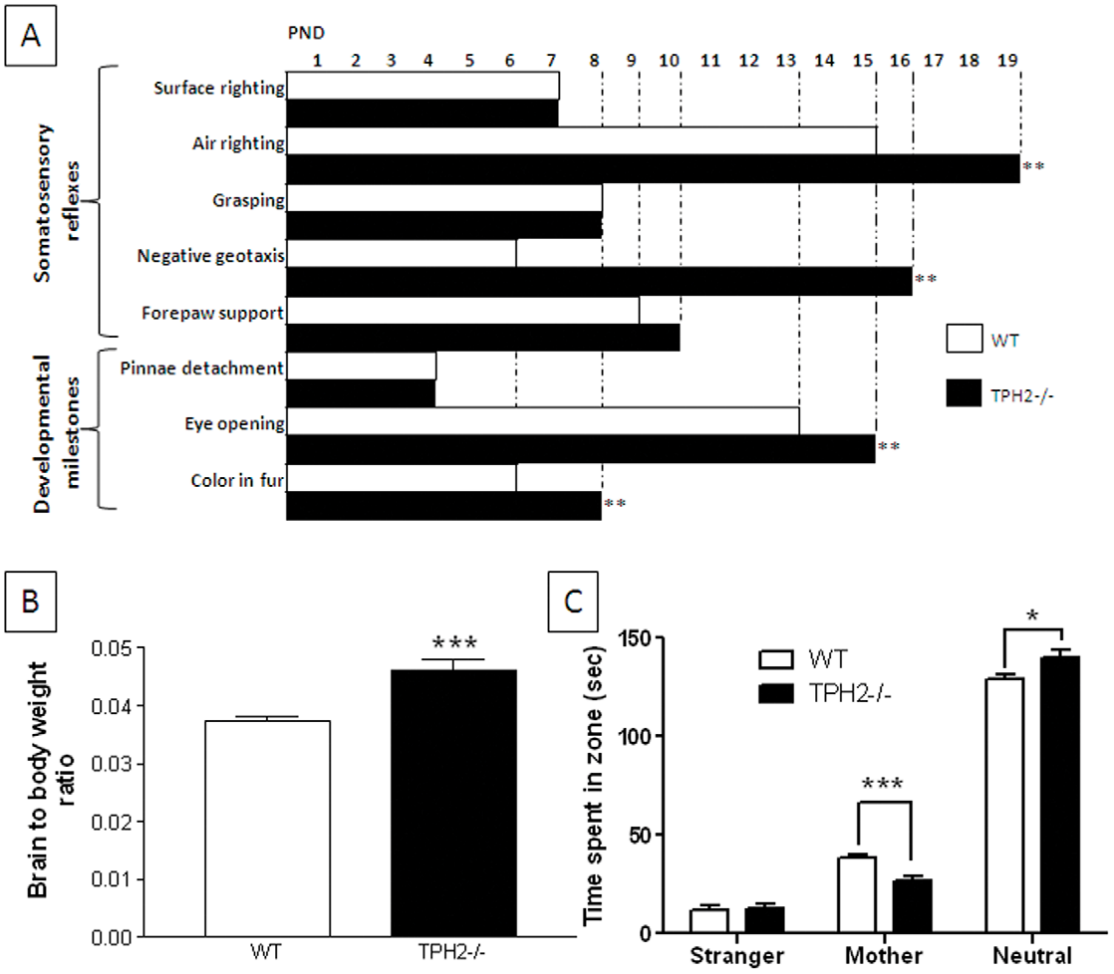
Developmental milestones, brain weight and social recognition in pre-weanling TPH2−/− pups. (A) acquisition of somatosensory reflexes and developmental milestones from PND 1–19. The indicated reflexes and milestones were considered acquired when they occurred for the second day in a row. (B) brain to body weight ratios in PND 25–28 mice and (C) maternal scent recognition in PND 14 mice. Data in panels (A) and (C) was collected from 6 independent litters for each genotype. Data included in panel (B) was based on results from 18 TPH2−/− (7 male, 11 female) and 31 WT controls (16 male, 15 female). The main effect of sex was not significant so data for males and females is combined. Data are presented as mean ± standard errors of the mean. Symbols indicate significant differences versus WT controls. * p<0.05; ** p<0.01; *** p<0.001.

TPH2−/− and WT pups were tested daily for the emergence of several somatosensory reflexes and developmental milestones and the results are presented in Figure 1A. The postnatal day (PND) representing the time of acquisition of the specific endpoint is indicated by the bars for each genotype. It can be seen that TPH2−/− mice are significantly delayed in acquisition of air righting (t(11) = 4.18 , p = 0.0015), negative geotaxis (t(12) = 3.67, p = 0.0032), eye opening (t(11) = 3.63, p = 0.0039) and coloration of fur (t(11) = 3.27. Figure 1B shows that the brain weight to body weight ratio was also significantly elevated in TPH2−/− mice (t(47) = 4.79, p<0.0001). TPH2−/− weanlings showed significantly lower body weights (t(47) = 5.17, p<0.0001) and brain weights (t(47) = 4.97, p<0.0001) by comparison to WT littermates (Figures S1A and S1B, respectfully). The differences in brain to body weight ratios were no longer apparent by 10–12 weeks of age (Figure S2). Because tests of socialization and repetitive behaviors in pre-weanling pups are limited in availability, we used the maternal scent test [57] as an indicator of social recognition in PND 14 mice. It can be seen in Figure 1C that TPH2−/− mice actually show significantly (t(10) = 4.82, p = 0.0007) lower preference for maternal scents by comparison to the scent of an unrelated (i.e., stranger) female. TPH2−/− mice also spend significantly (t(10) = 2.87, p = 0.0166) more time in the neutral zone of the test cage (i.e., litter not scented by another mouse) than WT controls. There was no indication that impaired movement capability was responsible for lower preference of maternal scents in the TPH2−/− mice (data not shown).

### Sociability in weanling TPH2−/− mice

#### Social memory

**Figure 2:**
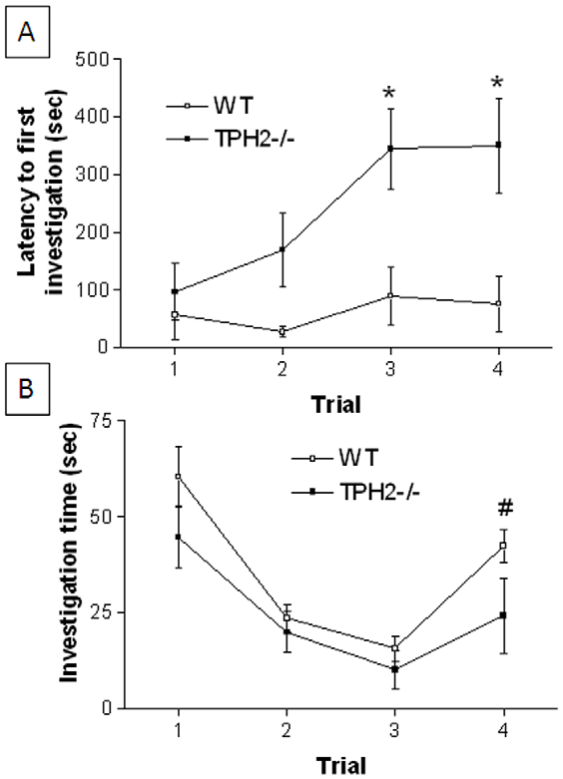
Social memory in weanling TPH2−/− mice. (A) latency to first investigate and (B) total time spent investigating the same stranger mouse on habituation trials 1–3 and a novel stranger on dishabituation trial 4. Data are presented as mean ± standard errors of the mean and are based on 12 TPH2−/− mice (6 males and 6 females) and 12 WT controls (6 males, 6 females). The main effect of sex was not significant so data for males and females is combined. Symbol indicates significant difference between WT and TPH2−/− (* p<0.025) or between habituation trial 3 and the dishabituation trial (# p<0.025).

The latency of WT mice to investigate a new cage mate is ∼1 min and remains fairly constant over all 3 habituation trials with a familiar mouse and following the introduction of a novel stranger mouse on dishabituation trial 4 as shown in Figure 2A. TPH2−/− mice show increasing latencies to investigate during the habituation and dishabituation trials (F_3,11_ = 3.40, p = 0.0292). This reduced interest in the familiar or a novel intruder reached latencies of 5–6 min in TPH2−/− but did not reach statistical significance. The main effects of genotype (F_1,3_ = 20.26, p<0.0001) and trial (F_1,3_ = 3.52, p = 0.018) were highly significant but the genotype X trial interaction was not (F_1,3_ = 1.90, p = 0.136). The time spent engaged in social investigation is presented in Figure 2B. WT mice show significantly reduced investigation of the familiar mouse over the 3 habituation trials (F_2,11_ = 32.53, p<0.0001) and investigation times increase significantly when a novel stranger mouse is introduced into the test cage on the dishabituation trial (p<0.025, Tukey's test). However, while TPH2−/− mice also show significant habituation over trials 1–3 (F_2,11_ = 7.21, p = 0.0039), they failed to show dishabituation in the presence of a novel stranger mouse (p>0.025, Tukey's test) although there was a trend toward an increase. The main effects of genotype (F_1,3_ = 5.85, p = 0.0177) and trial (F_1,3_ = 15.00, p<0.0001) were significant for TPH2−/− mice but the genotype X trial interaction was not (F_1,3_ = 0.67, p = 0.57).

#### Novel object test

**Figure 3:**
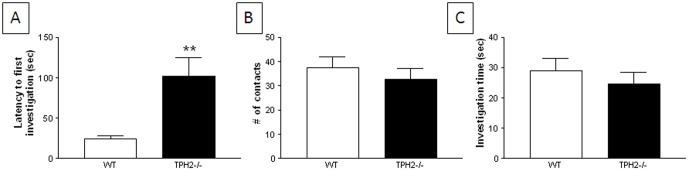
Novel object test of restricted interests in weanling TPH2−/− mice. (A) latency to first investigate, (B) total number of contacts and (C) total time investigating a novel object. Data are presented as mean ± standard errors of the mean and are based on 20 TPH2−/− mice (10 males, 10 females) and 22 WT controls (11 males, 11 females). The main effect of sex was not significant so data for males and females is combined. Symbols indicate significant differences from WT controls. ** p<0.01.

Figure 3A shows that weanling TPH2−/− mice have significantly (t(40) = 3.44, p = 0.0014) longer latencies to investigate a novel object by comparison to WT controls. TPH2−/− mice averaged just under 2 min to approach the object whereas WT mice do so within about 20 seconds. While TPH2−/− mice showed a trend for reduced number of contacts with the object (Figure 3B) and reduced total investigation time (Figure 3C), these measures did not reach statistical significance.

#### Social olfactory discrimination

**Figure 4:**
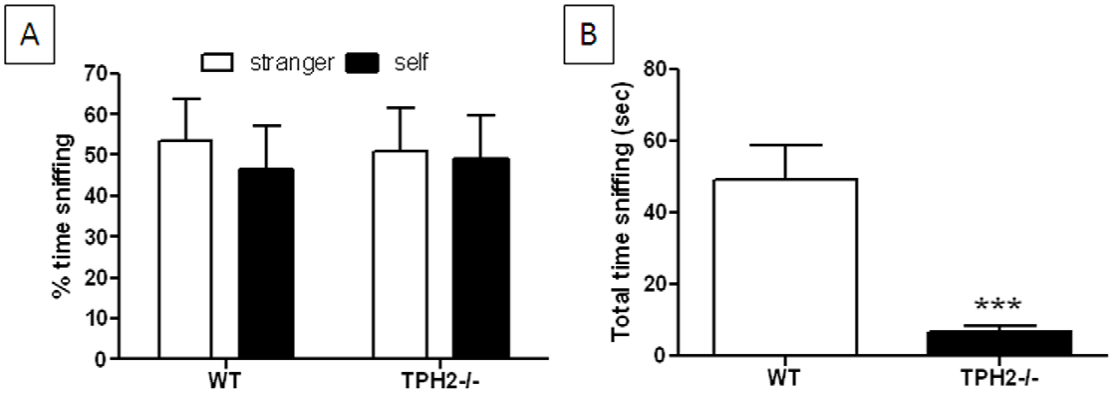
Social olfactory discrimination in weanling TPH2−/− mice. (A) % time spent sniffing litter scented by a stranger mouse versus that from test subject's own cage and (B) total time spent sniffing. Data are presented as mean ± standard errors of the mean and are based on 14 TPH2−/− mice (6 males, 8 females) and 13 WT controls (8 males, 5 females). The main effect of sex was not significant so data for males and females is combined. Symbols indicate significant differences from WT controls. *** p<0.001.

When exposed to objects permeated with the scent of their own litter versus that of a stranger, WT weanling mice presently showed a mild preference for the scent of a stranger over their own (53% versus 50% respectively) as shown in Figure 4A but this difference was not statistically significant. TPH2−/− weanling mice did not show a scent preference (Figure 4A) and actually spent significantly (t(25) = 4.64, p<0.0001) less time engaged in sniffing in this test by comparison to WT mice as shown in Figure 4B.

### Repetitive and compulsive behaviors in weanling TPH2−/− mice

**Figure 5:**
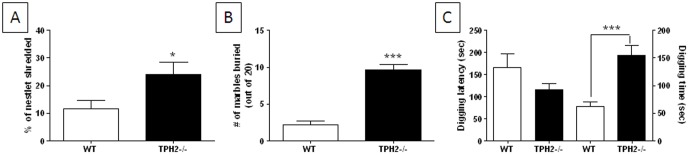
Repetitive behaviors in TPH2−/− weanling mice. (A) nestlet shredding based on 18 TPH2−/− mice (10 male, 8 females) and 21 WT controls (12 males, 9 females) , (B) number of marbles buried based on 23 TPH2−/− mice (12 males, 11 females) and 20 WT controls (10 males, 10 females), (C) latency to start digging (left y-axis) based on 20 TPH2−/− mice (10 males, 10 females) and 21 WT controls (11 males, 10 females) and total time spent digging (right y-axis) based on 20 TPH2−/− mice (10 male, 10 female) and 21 WT controls (11 male, 10 female). The main effect of sex was not significant for any test so data for males and females is combined. Data are presented as mean ± standard errors of the mean. Symbols indicate significant differences from WT controls. * p<0.025; *** p<0.001.

We have shown previously that adult TPH2−/− mice engage in high levels of repetitive and compulsive behaviors [48]. As such, the nestlet shredding and marble burying tests were used to assess these behavioral traits in TPH2−/− weanling mice. TPH2−/− mice shred significantly (t(37) = 2.40, p = 0.0214) more nestlet material (Figure 5A) and bury significantly (t(41) = 7.67, p<0.0001) more marbles (Figure 5B) than WT mice. We found previously that TPH2−/− mice show compulsive digging [48] so we assessed this behavior in weanlings as well. Figure 5C shows that TPH2−/− mice have a slightly shorter latency to commence digging once placed into the test cage by comparison to WT mice (left y-axis; p>0.05, Tukey's test) and they also spend significantly (t(39) = 4.55, p<0.0001) more time than WT mice engaged in digging (right y-axis; Figure 5C). We measured locomotor activity levels in these same cohorts of mice as described in Materials and Methods S1 and did not observe a genotype effect, ruling out a role for hyperactivity in influencing repetitive behaviors (Figure S3).

### Anxiety-like behaviors in weanling TPH2−/− mice

The light-dark and dark emergence tests of anxiety were used to test TPH2−/− weanling mice for anxiety-like behaviors as described in Materials and Methods S1. In the light-dark test, TPH2−/− weanling mice, like WT mice, spent more time in the dark chamber versus the light chamber and the allocation of time spent in each chamber was the same as WT controls (Figure S4A). Likewise, TPH2−/− mice emerged from the dark chamber into the light side of the light-dark box in the dark emergence test in the same amount of time as WT mice (Figure S4B).

### Sociability in adult TPH2−/− mice

#### Social memory

**Figure 6:**
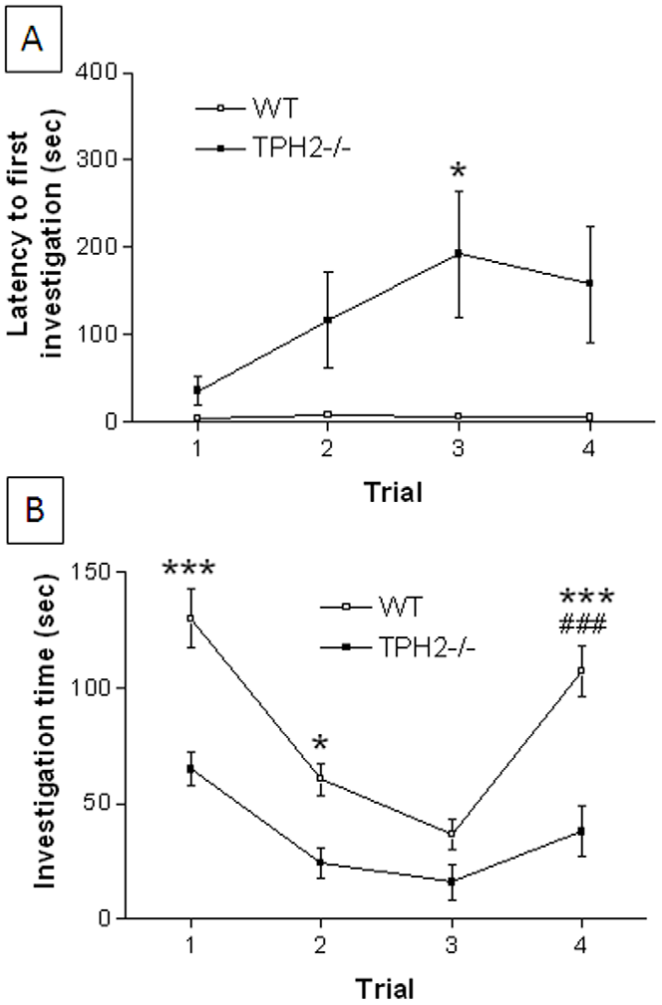
Social memory in TPH2−/− adult mice. (A) latency to first investigate and (B) total time spent investigating the same stranger mouse on habituation trials 1–3 and a novel stranger on dishabituation trial 4. Data are presented as mean ± standard errors of the mean and are based on 12 TPH2−/− mice (6 males and 6 females) and 12 WT controls (6 males, 6 females). The main effect of sex was not significant so data for males and females is combined. Symbols indicate significant difference between WT and TPH2−/− (* p<0.05, *** p<0.001) or between habituation trial 3 and the dishabituation trial (### p<0.001).

The latency of adult WT mice to investigate a new cage mate was less than 10 sec and did not vary significantly (F3,13 = 1.77, p = 0.17) over all 3 habituation trials with a familiar mouse and following the introduction of a novel stranger on dishabituation trial 4 as shown in Figure 6A. The latencies of TPH2−/− mice to investigate a new cage mate showed an increasing trend but did not reach significance (F_3,13_ = 2.42, p = 0.08). The main effect of genotype (F_1,3_ = 17.68, p<0.0001) was significant but neither the main effect of trial (F_1,3_ = 1.44, p = 0.2359) nor the genotype X trial interaction was significant (F_1,3_ = 1.38, p = 0.2535). The time spent in social interaction is presented in Figure 6B. WT mice show significantly reduced investigation of the familiar mouse over the 3 habituation trials (F_2,13_ = 57.29, p<0.0001) and investigation times increase significantly when a novel stranger mouse is introduced into the test cage on the dishabituation trial (p<0.001, Tukey's test). However, while TPH2−/− mice also show significant habituation over trials 1–3 (F_2,13_ = 31.23, p<0.0001), they failed to show dishabituation in the presence of a novel stranger mouse (p>0.05, Tukey's test). The main effects of genotype (F_1,3_ = 56.25, p<0.0001) and trial (F_1,3_ = 24.40, p<0.0001), and their interaction (F_1,3_ = 3.31, p = 0.0231) were significant.

#### Novel object test

**Figure 7:**
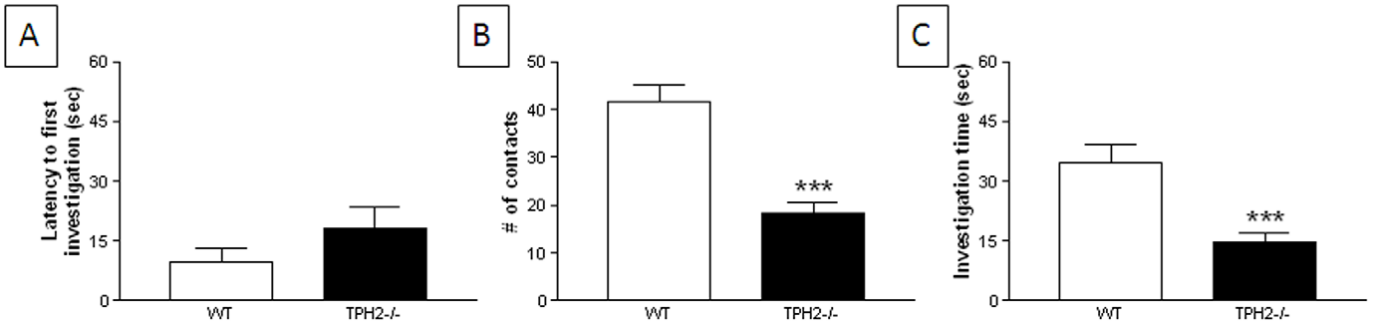
Novel object test of restricted interests in adult TPH2−/− mice. (A) latency to first investigate, (B) total number of contacts and (C) total time investigating a novel object. Data are presented as mean ± standard errors of the mean and are based on 20 TPH2−/− mice (10 males, 10 females) and 21 WT controls (11 males, 10 females). The main effect of sex was not significant so data for males and females is combined. Symbols indicate significant differences from WT controls. *** p<0.001.

TPH2−/− adults approached and investigated a novel object with the same latency of WT mice (Figure 7A). However, TPH2−/− mice showed much less interest in the object as revealed by significantly (t(39) = 5.51, p<0.0001) fewer numbers of contacts with the object (Figure 7B) and by spending significantly (t(39) = 4.00, p = 0.0003) less time in direct investigation of it (Figure 7C).

#### Social olfactory discrimination

**Figure 8:**
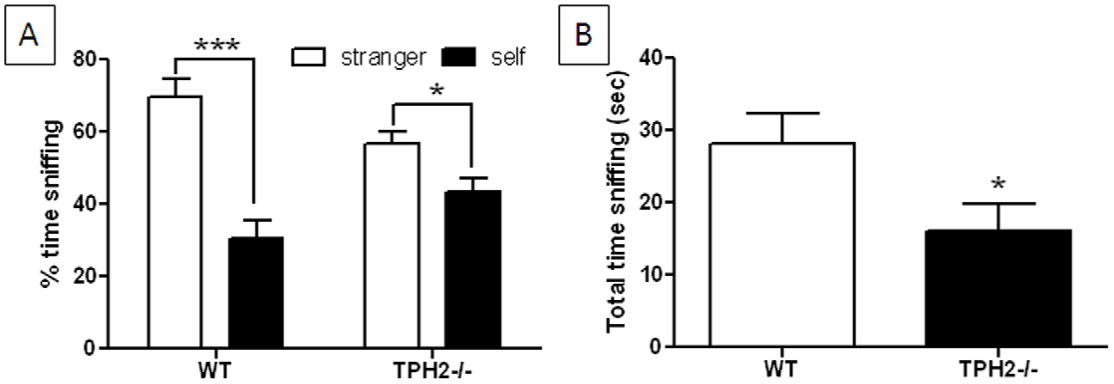
Social olfactory discrimination in adult TPH2−/− mice. (A) % time spent sniffing litter scented by stranger mouse versus that from test subject's own cage and (B) total time spent sniffing. Data are presented as mean ± standard errors of the mean and are based on 22 TPH2−/− mice (10 males, 12 females) and 21 WT controls (10 males, 11 females). The main effect of sex was not significant so data for males and females is combined. Symbols indicate significant differences from WT controls. * p<0.05; *** p<0.001.

In contrast to weanlings (see Figure 4 above), adult WT mice showed a significant (t(40) = 5.50, p<0.0001) preference for the scent of a stranger mouse over their own scent as shown in Figure 8A. TPH2−/− adults also showed a preference for the scent of a stranger mouse but the magnitude of this effect was slightly smaller than shown by WT mice (t(42) = 2.05, p = 0.0463). TPH2−/− mice also spent significantly (t(41) = 2.17, p = 0.0361) less time engaged in sniffing than WT controls (Figure 8B). In an attempt to rule out the possibility that TPH2−/− mice have a general olfactory abnormality, mice were tested in the odorant habituation test adopted from Wang and Storm [69] as described in Materials and Methods S1. Figure S5 indicates that WT (F_5,50_ = 4.97; p<0.0001) and TPH2−/− mice (F_5,50_ = 14.21, p<0.0001) spend significantly less time sniffing a non-social olfactory stimulus (water) over 5 trials. While each group also shows significant dishabituation (p<0.0001, Tukey's test for both genotypes), manifested as increased sniffing to a novel olfactory probe (vanilla extract) as compared to the last habituation trial, the main effect of genotype was not significantly significant (F_1,5_ = 0.39, p>0.05).

#### Direct social interaction

**Figure 9:**
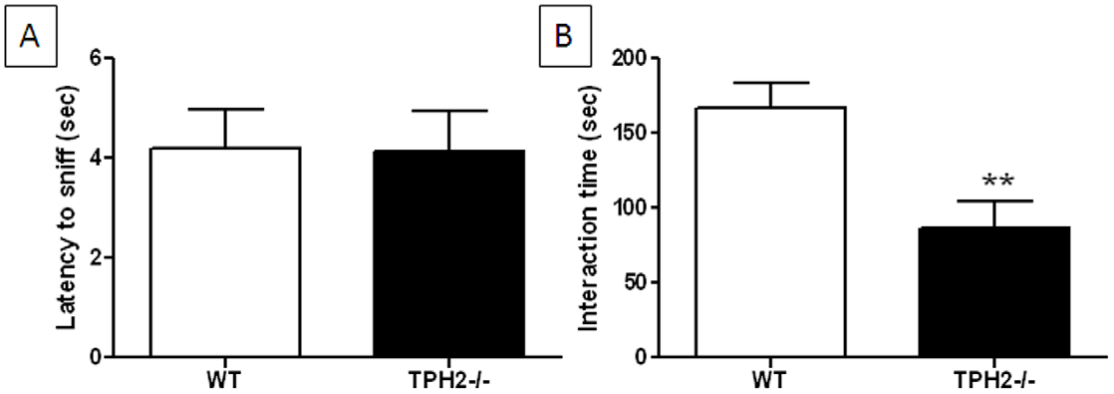
Direct social interactions in adult female TPH2−/− mice. (A) latency to sniff and (B) total interaction time. Data are presented as mean ± standard error of the mean and are based on 7 TPH2−/− mice and 11 WT controls and include only those TPH2−/− females that did not engage in attack behavior. Symbols indicate significant differences from WT controls. ** p<0.01.

We reported previously that TPH2−/− males and females are highly aggressive in one-on-one encounters [48], making it difficult to investigate reciprocal social interactions in mutant mice. Nevertheless, we attempted to study direct social interactions between adult WT and TPH2−/− females and observed that in 14 pairings of unfamiliar mice, 7 pairs did not engage in fighting and the results of their social interactions are presented in Figure 9. TPH2−/− mice were the same as WT controls in the latency to sniff an intruder (Figure 9A) but the mutant mice spent significantly (t(16) = 3.02, p = 0.0081) less time in direct interactions with the intruder mouse (Figure 9B). None of the 11 pairings of WT females resulted in aggressive behaviors in agreement with previous results in our lab [48].

#### Three chamber sociability test

**Figure 10:**
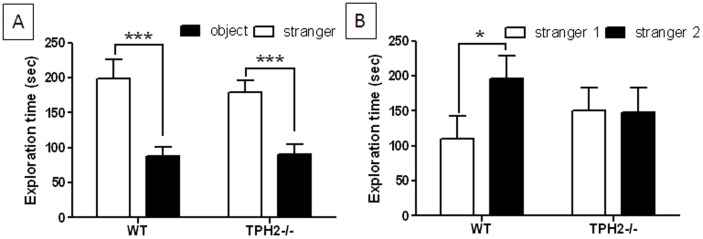
Three chamber sociability test in adult TPH2−/− mice. (A) exploration time in the sociability phase and (B) exploration time in the social novelty phase. Data are presented as mean ± standard error of the mean and are based on 14 TPH2−/− mice (7 males, 7 females) and 14 WT controls (7 males, 7 females). The main effect of sex was not significant so data for males and females is combined. Symbols indicate significant differences from WT controls. * p<0.05; *** p<0.001.

In the sociability phase of this test, TPH2−/− adult mice behaved in the same manner as WT controls, showing a preference for an animate (i.e., a stranger mouse confined within a cylinder) over an inanimate object (i.e., an empty cylinder) as shown in Figure 10A. However a significant difference emerged in the social novelty test phase. It can be seen in Figure 10B that when a new stranger mouse was introduced into the previously empty cylinder, WT mice show a significant (t(25) = 2.39, p = 0.0247) preference for a novel stranger mouse versus the now familiar stranger 1 mouse. In contrast, TPH2−/− mice did not show any preference for the new stranger mouse in this phase of the test, spending the same amount of time investigating the familiar and unfamiliar test subjects.

### Social communication in TPH2−/− adult mice

**Figure 11:**
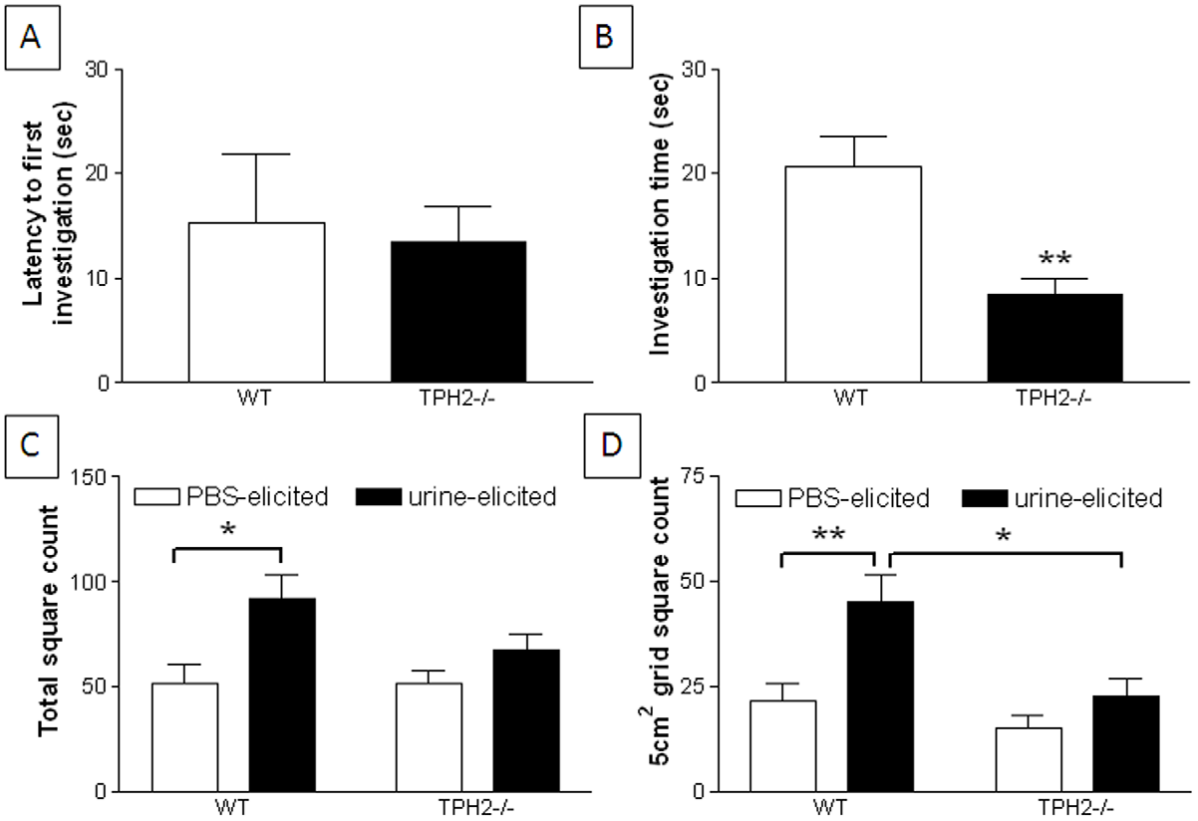
Urine scent marking in adult male TPH2−/− mice. (A) latency to first investigate urine spot from an estrous female, (B) time spent investigating urine spot from an estrous female, (C) number of urine deposits over the entire cage and (D) number of urine deposits in the immediate vicinity of the urine spot from an estrous female. Data are presented as mean ± standard error of the mean and are based on 7 TPH2−/− mice and 8 WT mice. Symbols indicate significant differences from WT controls. * p<0.05; ** p<0.01.

Urine scent marking was used as a measure of social communication in mice [65] and the results of this test are presented in Figure 11. Male TPH2−/− mice were not different from WT mice in the latency to investigate the scent of urine from a female mouse in estrous (Figure 11A). However, TPH2−/− mice spent significantly (t(18) = 3.47, p = 0.0041) less time sniffing the urine spot (Figure 11B) than WT. When urine deposits made by males in response to urine versus PBS were counted throughout the entire test cage, the results revealed that WT mice marked significantly (p<0.05, Tukey's test) more in response to urine (Figure 11C). The female urine spot did not elicit more scent deposits by TPH2−/− males than PBS as shown in Figure 11C. A count of all urine deposits made by males throughout the entire test cage cannot always discriminate between elicited versus spontaneous urination. Therefore, scent deposits were counted in the immediate vicinity (i.e., within a 5 cm^2^) of the urine test spot. It can be seen in Figure 11D that urine deposits by WT male mice remain significantly (p<0.01, Tukey's test) higher in number around the test urine spot versus PBS. TPH2−/− male urine deposits elicited by the female urine test spot were not different from deposits around PBS. Interesting, in this case, it can be seen that TPH2−/− mice deposited significantly fewer urinary markings (p<0.05, Tukey's test) in response to the test urine spot than did the WT males.

## Discussion

The present studies establish that mice lacking brain 5HT, resulting from a null mutation in the gene for TPH2, exhibit the core behavioral features of ASDs to include social interaction deficits, problems with social communication and repetitive behaviors. The behavior of the TPH2 knockout mice was cataloged at age periods corresponding to human early childhood (PND 1–21; pre-weanling), juvenile (PND 23–28; weanling) and adulthood (PND 10–12 weeks of age; [70]). Pre-weanling pups showed delays in acquisition of a number of somatosensory reflexes and developmental milestones, some of which are related to CNS function (e.g., negative geotaxis, air righting). These results agree well with another independent study that documented growth retardation in developing TPH2−/− mice [41]. TPH2−/− mice also show a transient brain overgrowth reflected as increased brain to body weight ratios at PND 25–28, an effect that normalizes as the mice reach adulthood. This finding is consistent with other studies in BALB/c mice that have reduced brain 5HT and similar ASD-like behavioral characteristics [71]. Large brain sizes have been associated with patients with ASD [72]. This brain overgrowth seems to occur during the first 2–3 years of life but does not persist until adulthood [73], [74], [75]. When exposed to a test chamber containing litter scented by a pup's mother or an unrelated female mouse, TPH2−/− mice showed less preference for the maternal scent than WT controls, suggesting early signs of a social communication deficit. TPH2−/− weanling mice were shown to have deficits in numerous tests of sociability and behavioral flexibility to include social memory, social olfactory discrimination and investigation of novel objects. TPH2−/− weanling mice show shorter periods of social investigation and reduced dishabituation when introduced into the presence of a stranger mouse by comparison to WT mice of the same age. Although TPH2−/− weanling mice did not show a clear preference for a novel scent versus their own in the social olfactory discrimination test, they were remarkable for their overall lack of interest in either scent. This agrees with what was observed in pre-weanling TPH2−/− mice in the maternal scent test. TPH2−/− weanling mice also showed significant repetitive behaviors in the form of nestlet shredding, marble burying and compulsive digging all of which persisted into adulthood. These repetitive behaviors in weanling mice were not confounded by anxiety-like behaviors or changes in the levels of locomotor activity.

In agreement with Alenina et al. [41] and Mosienko et al. [51], we have already shown that adult TPH2−/− mice display significantly increased levels of aggression, another indication of a social interaction deficit [48]. Presently, we show that the sociability deficits in weanling TPH2−/− mice (i.e., social memory, social olfactory discrimination and restricted interests) persist into adulthood and are accompanied by other problems with socialization as well. First, female TPH2−/− mice that were not aggressive in the presence of a conspecific WT female (7 of 14 pairings did result in aggression) spent significantly less time in direct social interaction by comparison to controls. Second, in the three chamber sociability test, TPH2−/− adults showed normal preference for a stranger mouse over an inanimate object but they showed no preference for a novel mouse by comparison to a familiar mouse in agreement with the outcomes of other social interaction tests (see above). This lack of preference for social novelty displayed by TPH2−/− mice in the three chamber sociability test is similar to that of other mouse genetic models of ASDs to include NL4 [76], NL3 [77], Gabrb3 [78], SLC6A4 [52] and Fgf17 [79]. Because olfaction is the primary mode of communication in mice [65] and is not impaired in TPH2−/− mice [48 and Figure S5], we used urine scent marking as an indicator of social communication in TPH2−/− mice. It was observed that male TPH2−/− mice deposited significantly fewer scent marks in response to urine from females in estrous. This trait was even more evident when markings in the immediate vicinity of the female scent spot were measured where it could be shown that TPH2−/− mice deposited significantly fewer scent marks than WT controls. The number of urine deposits made by TPH2−/− mice was the same as WT mice in response to non-urine stimuli, indicating that the TPH2−/− mice were not deficient in urinary function and that they were indifferent to the socially relevant urinary stimulus. Ultrasonic vocalizations (USVs) are another form of social communication used by rodents [80], [81] and which may have utility in assessing communication deficits in mouse models of ASD (e.g. see [66]). We did not assess USVs currently but it has been shown that TPH2−/− neonates appear to emit vocalizations upon maternal separation that are the same as WT newborns [41]. Additional study of USVs by TPH2−/− mice are called for to expand on the early observations of Alenina et al. [41] and to determine if this other form of communication is impaired in mice lacking brain 5HT. Other behaviors that could have a confounding influence on the social behaviors of adult mice measured presently, such as anxiety-like behaviors, alterations in balance and coordination, and changes in locomotor activity have been shown to be normal in TPH2−/− mice (see Figure S3 and [48]). Mosienko and colleagues also showed that TPH2−/− mice have decreased anxiety-like behavior [51]. While TPH2−/− mice are very aggressive, we do not view this as a confounding behavior but one that is also characteristic of ASDs [71], [82], [83], albeit not a core behavioral trait.

It is certainly not the case that individuals with ASDs have a total lack of brain 5HT, but the consequences of a dysfunction in 5HT neurochemistry during critical periods of CNS development could be revealed in the complex, heterogeneous behavioral phenotype of this disorder. A large body of evidence has accumulated in support of an extremely important role for 5HT in the development of the brain [27], [28], [29], [30], [32]. For instance, 5HT signaling is thought to direct such diverse processes as neurogenesis, synaptogenesis, corticogenesis, neuronal migration and maturation, and axonal network formation [30], [32], [33], [35], [36], [37]. The expression patterns of selected 5HT receptors during development overlap with receptors involved in axonal guidance [84] and 5HT signaling determines the specific trajectory of thalamocortical pathways via its influence on axonal responses to the axon guidance molecule netrin-1 [85]. The developing forebrain may also depend on an exogenous source of 5HT. At early stages of brain development prior to the time when 5HT is supplied to the forebrain endogenously by emerging projections from the 5HT neurons of the dorsal raphe (e.g., embryonic days 10–11 in mice), 5HT can be supplied by the placenta [31]. The fact that an extraneous source of 5HT can influence the developing fetal brain serves as further testament to the importance of this monoamine in neurodevelopment [31], [32] and establishes the possibility that disruptions in 5HT function at the level of the placenta or within the CNS can have enduring behavioral consequences. It is also well established that maternal 5HT (i.e., non-neuronal from TPH1) is essential for murine morphogenesis and embryonic development [86]. Thus, while ASDs are being viewed increasingly as disorders of the synapse [87] or misconnection syndromes resulting from altered gene expression [38], the possibility remains that early disruptions in 5HT function during critical CNS development periods could alter brain wiring resulting in persistent effects on postnatal behaviors. For these reasons, the TPH2−/− mouse model constitutes a valuable resource for investigating how brain network formation and synaptic gene expression are influenced by a lack of endogenous 5HT during development, particularly as it relates to ASDs.

Mouse genetic models of ASDs are contributing valuable mechanistic information on those factors that can alter susceptibility to this disorder [88], [89], [90]. Apart from strains with targeted disruptions in genes that contribute to ASD susceptibility in humans (e.g., see [90] for review), inbred mouse strains show considerable heterogeneity in social behavior. Some of these strains including BALBc and BTBR T+tf/J are on the low end of the sociability continuum and are used frequently as models of ASDs [71], [91], [92], [93]. Of relevance to our current studies, 129Sv mice generally score between the extremes of high (e.g., FVB) and low sociability (e.g., BTBR T+tfJ; [91], [92]). Our TPH2−/− mice are on a mixed C57BL/6-129Sv background but only mice lacking the gene for TPH2−/− show behaviors relevant to ASDs by comparison to their WT littermates. This observation leads us to conclude that the lack of 5HT, not the background strain of our mice, is responsible for ASD-like behaviors. It is also interesting and relevant that those inbred mouse strains showing many of the core behavioral deficits associated with ASD have 5HT deficiencies. BTBR T+tf/J mice have mild reductions in 5HT neurochemical function [94] and respond to drugs that enhance 5HT function with increases in sociability [95], [96]. BALB/c mice have a single nucleotide polymorphism in the TPH2 gene which results in a ∼50% reduction in brain TPH2 catalytic function and 5HT levels [97]. BALB/c mice show numerous ASD-like behaviors [71], [91], [92] and high levels of aggression [98] as seen in TPH2−/− mice [41], [48], [51]. The present results showing that mice lacking brain 5HT exhibit some of the core behavioral deficits of ASDs adds support to the possibility that diminished 5HT neurochemical function in at least BTBR T+tf/J and BALB/c mice underlies the low sociability and ASD-like behaviors known to exist in these strains.

We reported recently that TPH2−/− mice were characterized with a phenotype of behavioral disinhibition [48]. Our observations that TPH2−/− mice showed high levels of compulsive and impulsive behaviors is completely consistent with current behavioral analyses relating to ASD-like behaviors. In fact, OCD-like behaviors are common in children with ASDs [99], [100]. The present behavioral phenotyping of TPH2−/− mice focused on social behaviors and extended characterization of these mice as neonates, pre-weanlings (1–21 days of age), weanlings (23–28 days of age) and adults. It is clear that neurodevelopment delays and ASD-like behaviors are evident in very young TPH2−/− mice and these behavioral abnormalities persist through adolescence and into adulthood. The present observations of ASD-like behaviors in TPH2−/− mice are novel and suggest that these mice may be on the deficient end of a continuum of sociability. Despite the fact that so many different groups have independently developed rodent models with mutations in the gene for TPH2 [41], [42], [43], [44], [45], [46], [47], [48], [49], [50], and considering the link of low TPH2 function to ASD [7], [9], [10], it is very surprising that only three groups have reported behaviors in these mice with relevance to ASDs to include aggression [41], [48], [51] and impaired maternal care [41]. Mice lacking the gene for Pet-1 have a partial knockdown of 5HT levels, due to a reduction in the number of 5HT neurons, and show social deficiencies in the form of poor maternal care and aggression [101]. Animals lacking the gene for the SERT [52] or expressing a SERT gain-of-function coding variant seen in ASD [53] show social impairments, and targeted disruption of the 5HT1C receptor gene results in compulsive behaviors [54]. Mice carrying a duplicated chromosome 7C (orthologous to human chromosome 15q11-13), a copy number variant associated with ASD-like behaviors [102], are now known to have significantly lowered 5HT levels [103]. The present results, when considered next to other mouse models with genetic disruptions that impact the 5HT neuronal system, strongly suggest that hyposerotonemia is associated with ASD-like behaviors.

Our present results suggest that TPH2−/− mice could serve as a useful model to study ASDs and particularly those forms with functional 5HT deficits. Mice lacking brain 5HT exhibit some of the behavioral deficits of ASDs but like all other existing animal models, there are limitations that may restrict its use as a generalized model of ASDs. For example, the ASDs show a frequency bias in males [104], [105] whereas we did not observe a male-dominance for ASD-like behaviors in the TPH2−/− mice. Weanling TPH2−/− mice displayed neither restricted interests nor deficits in social olfactory discrimination. The lack of a deficit in USVs in TPH2−/− mice also suggests that at least this one form of communication is normal [41]. Despite these limitations, the TPH2−/− mouse could serve as a valuable model of ASDs from a neurodevelopmental perspective. In any case, we should stress that the focus of the present work is on 5HT, and not TPH2 per se, and how a lack of 5HT can contribute to ASD-like behaviors. A large number of psychiatric illnesses have been associated with polymorphisms in the TPH2 gene to include depression, anxiety and obsessive-compulsive disorder [106]. Studies of an association of TPH2 genetic variants with ASDs are suggesting that TPH2 may not be an ASD susceptibility gene [18], [19], [20], [21]. However, changes in the function of its protein product can have broad effects on 5HT synthesis. In light of the importance of 5HT production during development on the normal formation of brain pathways and networks, a change in TPH2 activity during this critical period, even short term, could have long-lasting effects on brain development and subsequent behavioral outcomes. The challenge of future research will be to determine if the 5HT deficiency responsible for ASD-like behaviors arises during prenatal development or postnatally.

## Supporting Information

Figure S1 **Brain weight and body weight determinations of TPH2−/− mice.** (A) mice at PND 25–28 were weighed and immediately decapitated and (B) whole brains (from the rostral pole to the cervicomedullary junction) were dissected from the skull and weighed on a precision balance. Data are presented as mean ± standard error of the mean and are based on 18 TPH2−/− mice (7 male, 11 female) and 31 WT controls (16 male, 15 female). The main effect of sex was not significant so data from males and females is combined. *** p<0.0001.
(TIF)

Figure S2 **Brain to body weight ratios of adult TPH2−/− mice.** (A) body weight, (B) brain weight and (C) brain to body weight ratios for adult (10–12 weeks of age) mice. Data are presented as mean ± standard error of the mean and are based on 16 TPH2−/− mice (11 male, 5 female) and 19 WT controls (14 male, 5 female). The main effect of sex was not significant so data from males and females is combined. * p<0.05.
(TIF)

Figure S3 **Locomotor activity in weanling TPH2−/− mice.** Spontaneous locomotor activity was measured in TPH2−/− mice (N = 21; 11 male, 10 female) and WT controls (N = 27; 13 male, 14 female) for 30 min in an automated locomotor activity chamber. Data are presented as mean ± standard error of the mean. The main effects of genotype and sex were not significant so data from males and females is combined.
(TIF)

Figure S4 **Anxiety-like behaviors in weanling TPH2−/− mice.** (A) time spent in the light and dark compartments and (B) time to emerge from the dark compartment into the light compartment of a light dark box. Data are mean ± standard error of the mean and are based on 22 TPH2−/− mice (11 male, 11 female) and 22 WT controls (12 male, 10 female). The main effects of genotype and sex were not significant on either test so data from males and females is combined.
(TIF)

Figure S5 **Odorant habituation test of olfactory acuity in adult TPH2−/− mice.** Mice of both sexes and genotypes were placed into a cage containing an olfactory stimulus (water-laced kimwipe) for 5 habituation trials followed by the introduction of a new olfactory stimulus (vanilla extract-laced kimwipe) in a single dishabituation trial. Time spent sniffing during the habituation and dishabituation trials was recorded and data are presented as mean ± standard error of the mean and are based on 11 TPH2−/− (6 males and 5 females) and 11 WT mice (6 males and 5 females). The main effect of sex was not significantly different so data from males and females is combined. The main effect of genotype was also not significant. Symbols indicate a significant difference between habituation trial 5 and the dishabituation trial (### p<0.0001 for WT; δδδ p<0.001 for TPH2−/−).
(TIF)

Materials and Methods S1 Mice Genetically Depleted of Brain Serotonin Display Social Impairments, Communication. Deficits and Repetitive Behaviors: Possible Relevance to Autism.
(DOC)
