# Supplementary figures and images for: Mice Genetically Depleted of Brain Serotonin Display Social Impairments, Communication Deficits and Repetitive Behaviors: Possible Relevance to Autism
Source: PLoS One. 2012 Nov 6;7(11):e48975. doi: 10.1371/journal.pone.0048975 (PMC3490915; doi:10.1371/journal.pone.0048975)

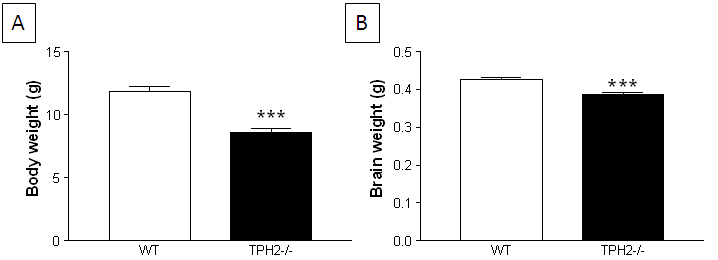

Supplement: Figure S1 — Brain weight and body weight determinations of TPH2−/− mice. (A) mice at PND 25–28 were weighed and immediately decapitated and (B) whole brains (from the rostral pole to the cervicomedullary junction) were dissected from the skull and weighed on a precision balance. Data are presented as mean ± standard error of the mean and are based on 18 TPH2−/− mice (7 male, 11 female) and 31 WT controls (16 male, 15 female). The main effect of sex was not significant so data from males and females is combined. *** p<0.0001. (TIF) [file pone.0048975.s001.tif]

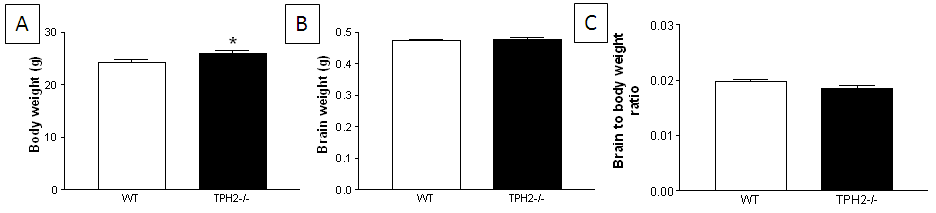

Supplement: Figure S2 — Brain to body weight ratios of adult TPH2−/− mice. (A) body weight, (B) brain weight and (C) brain to body weight ratios for adult (10–12 weeks of age) mice. Data are presented as mean ± standard error of the mean and are based on 16 TPH2−/− mice (11 male, 5 female) and 19 WT controls (14 male, 5 female). The main effect of sex was not significant so data from males and females is combined. * p<0.05. (TIF) [file pone.0048975.s002.tif]

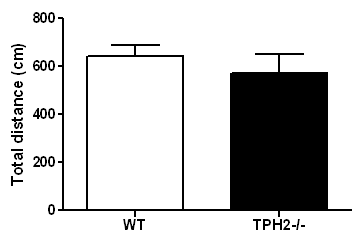

Supplement: Figure S3 — Locomotor activity in weanling TPH2−/− mice. Spontaneous locomotor activity was measured in TPH2−/− mice (N = 21; 11 male, 10 female) and WT controls (N = 27; 13 male, 14 female) for 30 min in an automated locomotor activity chamber. Data are presented as mean ± standard error of the mean. The main effects of genotype and sex were not significant so data from males and females is combined. (TIF) [file pone.0048975.s003.tif]

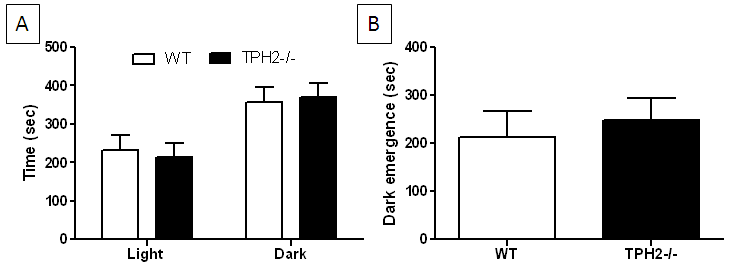

Supplement: Figure S4 — Anxiety-like behaviors in weanling TPH2−/− mice. (A) time spent in the light and dark compartments and (B) time to emerge from the dark compartment into the light compartment of a light dark box. Data are mean ± standard error of the mean and are based on 22 TPH2−/− mice (11 male, 11 female) and 22 WT controls (12 male, 10 female). The main effects of genotype and sex were not significant on either test so data from males and females is combined. (TIF) [file pone.0048975.s004.tif]

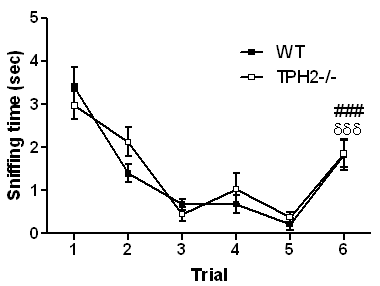

Supplement: Figure S5 — Odorant habituation test of olfactory acuity in adult TPH2−/− mice. Mice of both sexes and genotypes were placed into a cage containing an olfactory stimulus (water-laced kimwipe) for 5 habituation trials followed by the introduction of a new olfactory stimulus (vanilla extract-laced kimwipe) in a single dishabituation trial. Time spent sniffing during the habituation and dishabituation trials was recorded and data are presented as mean ± standard error of the mean and are based on 11 TPH2−/− (6 males and 5 females) and 11 WT mice (6 males and 5 females). The main effect of sex was not significantly different so data from males and females is combined. The main effect of genotype was also not significant. Symbols indicate a significant difference between habituation trial 5 and the dishabituation trial (### p<0.0001 for WT; δδδ p<0.001 for TPH2−/−). (TIF) [file pone.0048975.s005.tif]
