# Supplementary material for: Mice Genetically Depleted of Brain Serotonin Display Social Impairments, Communication Deficits and Repetitive Behaviors: Possible Relevance to Autism
Source: PLoS One. 2012 Nov 6;7(11):e48975. doi: 10.1371/journal.pone.0048975 (PMC3490915; doi:10.1371/journal.pone.0048975)
Supplement: Materials and Methods S1 — Mice Genetically Depleted of Brain Serotonin Display Social Impairments, Communication. Deficits and Repetitive Behaviors: Possible Relevance to Autism. (DOC) [file pone.0048975.s006.doc]

**Supporting Materials and Methods, Kane et al., Mice Genetically Depleted of Brain Serotonin Display Social Impairments, Communication Deficits and Repetitive Behaviors: Possible Relevance to Autism**

**Brain to body weight ratios**. TPH2-/- and WT mice at the ages of PND 25-28 and 10-12 weeks were weighed and immediately decapitated. Whole brains (from the rostral pole to the cervicomedullary junction) were dissected from the skull and weighed on a precision balance.

**Spontaneous locomotor activity.** Spontaneous locomotor activity was measured for 30 min in an open field-locomotor activity apparatus (AccuScan Instruments, Columbus, OH).

**Light-dark preference test of anxiety.** The test was performed as previously described [1]. Briefly, mice were placed into the light compartment of the open field-locomotor activity apparatus (AccuScan Instruments) facing away from the dark chamber. The number of entries into the dark chamber, the total time spent in each compartment, and total distance traveled were recorded for each mouse in 10 min sessions.

**Dark emergence test of anxiety.** The test was performed as previously described [1]. Briefly, mice were placed into the dark compartment of the open field-locomotor activity apparatus (AccuScan Instruments) facing away from the opening between compartments. The time to emerge fully (i.e., all 4 paws) into the lighted compartment (up to 10 min) was measured.

**Odorant habituation test of olfactory acuity**. This test was adapted from Wang and Storm [2]. Briefly, WT and TPH2-/- mice were placed individually into a small polypropylene cage with no bedding. A folded kimwipe was placed inside a low-density polyethylene capsule (2.5 cm in length) with both ends open. The kimwipe was laced with 50 µl of water and immediately introduced into the cage. The amount of time the subject spent sniffing the capsule was recorded in 2 min trials. This procedure was repeated 5 times with 1 min intertrial intervals. After the 5^th^ habituation trial, a new capsule containing a folded kimwipe laced with 25 µl of vanilla extract was introduced and the time spent sniffing the new stimulus was recorded for 2 min (i.e., dishabituation). Mice were removed from the test cage between all trials.

**Statistical Analysis**. Student’s 2-tailed t-tests were performed to analyze the data corresponding to brain to body weight ratios, locomotor activity, light-dark preference and dark emergence. p values < 0.05 were deemed statistically significant. A 2-way ANOVA was used to analyze data from the odorant habituation test in adult mice for the main effects of genotype and trial. Repeated measures ANOVAs followed by Tukey’s test were performed to analyze habituation and dishabituation trials within each genotype. In those cases where cohorts of mice were used in two tests, Bonferroni’s correction was applied to control for the familywise error rate and the p value for significance was set to 0.025. All statistical analyses were carried out using GraphPad Prism version 5.04 for Windows, GraphPad Software, San Diego, CA, www.graphpad.com.

1. Angoa-Peréz M, Kane MJ, Briggs DI, Sykes CE, Shah MM, et al. (2012) Genetic depletion of brain 5HT reveals a common molecular pathway mediating compulsivity and impulsivity. J Neurochem 121: 974-984.

2. Wang Z, Storm DR (2011) Maternal behavior is impaired in female mice lacking type 3 adenylyl cyclase. Neuropsychopharmacology 36: 772-781.
